# Loot

**Published:** 1868-01-01

**Authors:** 


### *LOOT.*

---

The *Galveston Medical Journal* (Prof. G. Dowell), recommends in cases of *poisoning by Rhus Toxicodendron*, and other poisonous species of the Rhus, to bathe the parts with a solution of caustic potash, sufficiently strong to render soapy the skin. This "has never failed to cure it immediately," although he has used it in hundreds of cases, including himself. The potash is used in the proportion of ten grs. to the ounce of water, but may be increased in strength as needed. A stronger solution will relieve the effects of the same poison upon the skin of animals.

F. B. Greenough, M. D., of Boston (B. M. & S. Jour.), in an article on *Gonorrhœal Rheumatism* observes: "With regard to treatment, perfect rest is the most essential part. If the pain is severe, two or three leeches may be applied with relief. Evaporating lotions may also be tried. When there is an effusion into the joint, the part may be rubbed with a salve containing iodine or iodide of potassium. If resorption does not take place, compression should be used, either by bandages, or still better by compressed sponges. All authorities agree that the remedies which have been supposed to be beneficial in rheumatism, such as the alkalies, colchicum, iodide of potassa, etc., have no influence in this affection. Perhaps it is lucky for us that the differential diagnosis is not dependent on this fact alone. The urethral trouble must be treated exactly the same as it would be if no articular complication existed."

A correspondent of the same journal furnishes an abstract of a paper before the French Academie Imperiale de Medicine, by Prof. Semola of Naples, on *Bright's Disease*, which concludes: "According to this author, Bright's Disease is not the result of a primitive anatomical lesion of the kidneys, but is a result of the double series of effects [prevention of the oxidation of the materials introduced into the system in the form of peptones (the products of the digestion of albumen), and resulting congestion of the viscera, especially of the kidneys] which succeed the more or less sudden suppression of the functions of the skin. The aim of the physician should be to re-establish these functions, and thus increase oxidation of the peptones, and relieve the renal congestion. Among the means best suited to this purpose are the ordinary sweatings, or, in obstinate cases, hot air baths, always followed by cool or cold shower baths; preparations of arsenic, and inhalations of oxygen. The diet should be vegetable or starchy, with but very little meat."

The *Abortive Treatment of Typhoid Fever* by repeated blistering over the iliac region is revived by Dr. Strong of Buffalo, and the editor of the *Pacific Medical Journal* is inclined to concur with him. The latter observes that this method has been employed by other California physicians. He also suggests that other topical means may be resorted to.

The *Brown Cod Liver Oil*, it is still insisted by some, is the most efficient because it contains the most biliary matter.

Maisonneuve treats *Mammary Cancer* by a new method. He passes a bistoury into the tumor, and afterward inserts slips ("arrows") of dried chloride of zinc and flour into the cavity, where they are allowed to remain and gradually dissolve, this usually requiring about ten days. The slips are made by mixing equal parts of the chloride and flour. The paste thus formed is dried, and the slips cut of the size desired. The pain is said not to be very great, and the effect is to kill the tumor by mummifying the tissues. The process is designated *cauterization en fleche*, and seems to have points of superiority over that adopted by the colored gentleman whose exploits in cancer curing a few years since are still remembered in Paris and elsewhere.

Dr. Durkee (B. M. J.) recommends common soft soap, well rubbed in, as the best application for *Scabies.*

*Acetic Acid* injections into *Cancerous Tumors*, according to the report of two cases by Fred. D. Lente, M. D., of Cold Spring, N. Y., (N. Y. Med. J., Dec., 1867,) do not seem to sustain the encomiums lavished upon them across the sea. Though great care was taken, and the cases themselves favorable, the result was failure in each.

*Permanganate of Potash*, gr. ss to the ounce of water, is recommended strongly as a gargle in Diphtheria. "Its use should be accompanied by ferruginous tonics, wines and careful nourishment." True. *Carbolic Acid* is also commended for this purpose.

Mr. Charles Sedgwick, in the *Medical Times and Gazette*, prescribes the following form: ℞ Acid, Carbolic Mxx; acidi acetici, half a fluid drachm; mellis, two drams; tinct. myrrhæ, two fluid drachms; aquae q. s. to six fluid ounces; mix and make a gargle. The carbolic and acetic acids to be well shaken together; the honey to be added to the water gradually. Internally tincture of iron and quinine."

Dr. Lortet (*Reporter Periscope*) has administered in a few cases of *Tape Worms*, which had resisted the usual remedies, *Sulphuric Ether*, which apparently acted upon them as it does on man, as an anæsthetic, rendering them insensible, when they are easily removed by a mild purgative. The plan is to give five drachms of ether at a dose, and to follow it in two hours by an ounce of castor oil. The worm is discharged without causing pain, entire or almost so, and always with the cephalic end intact. Though but few have been thus treated, the practice is worth bearing in mind.

Quite a range is it in the treatment of *Gonorrhea* from argent. nitrat. forty grs. to the ounce, down to the latest proposed lavement, which is finely powdered starch mixed with lukewarm water to the consistence of cream, but thin enough for injection. Yet M. Luc (*Lond. Lancet*), military surgeon, finds it excellent either in painless discharges or when the inflammatory stage is over.

*Nutrition.—Dr. Lionel Beale* believes that the serum of the blood is the nutritive pabulum of the body; that the red corpuscles are concerned in its distribution and in preventing changes in the composition of the great mass of the blood as certain constituents are removed from or poured into it; that the white corpuscles are masses of germinal matter concerned in the formation of the serum as well as of the red corpuscles; and that the special products of nutrition depend not so much on the characters of the pabulum as upon the *converting* powers of the germinal matter throughout the textures and which *appropriates* from the pabulum the materials it requires. The red corpuscles have therefore assigned to them a secondary position as agents of nutrition. The principal argument in support of this is derived from the fact, that elaborate tissues are formed in animals which have no colored blood corpuscles. According to Beale, three distinct phenomena are involed in nuitrition. 1. The contact of the soluble pabulum with the germinal matter of the tissues. 2. The separation of the elements of the pabulum from their state of combination. 3. The rearrangement of these elements and the conversion of some of them into new germinal matter.—*Journ. Anat. and Phys.*, Nov. 1867, from *Quarterly Jl. of Microscop. Sc.*, July, 1867.

*Pepsine in Vomiting of Pregnancy.—M. Gross* has used pepsine in doses of eight grains before meals, with complete relief, in a case of obstinate vomiting in pregnancy.

